# Risk factors for acquisition of ventilator-associated pneumonia in adult intensive care units

**DOI:** 10.12669/pjms.295.3375

**Published:** 2013

**Authors:** Fariba Lahoorpour, Ali Delpisheh, Abdorrahim Afkhamzadeh

**Affiliations:** 1 Fariba Lahoorpour, PhD candidate of Bacteriology, Department of Pathology and Medical Laboratory Sciences, Faculty of Para Medicine, Kurdistan University of Medical Sciences, Sanandaj, Iran.; 2 Ali Delpisheh, PhD, PostDoc, Professor of Clinical Epidemiology, Department of Epidemiology & Prevention of Psychosocial Injuries Research Centre, Ilam University of Medical Sciences, Ilam-Iran.; 3 Abdorrahim Afkhamzadeh, MD, MPH, Assistant Professor of Community Medicine, Department of Community Medicine, Faculty of Medicine, Kurdistan Research Center for Social Determinants of Health, Kurdistan University of Medical Sciences, Sanandaj, Iran.

**Keywords:** Ventilator-Associated Pneumonia (VAP), Intensive Care Unit

## Abstract

***Objective:*** Ventilator Associated Pneumonia (VAP) has an imperative place amongst nosocomial infections leading to increase morbidity and mortality rates. The present study aimed to determine risk factors for acquisition of ventilator- associated pneumonia in an intensive care unit (ICU).

***Methods:*** A nested case-control study was carried out from September 2007 to June 2008. All 183 patients hospitalized at the adult ICU ward in Be’sat Hospital, Sanandaj city western Iran over a 48 hour period were included. Bacteriologic diagnosis and antibiotic susceptibility patterns were performed based on Edward & Ewing’s methods and CLSI system guidelines.

***Results*** *:* Of the 149 samples which were taken from endotracheal tubes of 183 patients, 48 cases were diagnosed for VAP with an incidence rate of 26.2%. Mean duration of hospitalization was 23.4±10.2 days. The maximum and minimum antibiotic resistance for the gram negative bacteria was 93.3% for *Cefalotin* and 50% for *Amikacin*. The main risk factors for acquisition of ventilator- associated pneumonia were mechanical ventilation (Adjusted OR: 1.55, 95% CI: 1.37-1.74), history of antibiotic consumption (AOR: 8.92, CI: 1.16- 66.66) and fever (AOR: 3.11, CI: 1.22- 7.93).

***Conclusions:*** VAP is significantly related to ICU hospitalization, mechanical ventilation and history of antibiotics consumption. *Cefalotin* and *Amikacin* showed the highest and lowest antibiotic resistance against gram negative bacteria respectively.

## INTRODUCTION

Ventilator-associated pneumonia (VAP) occurs almost 48 hours after the initiation of endotracheal intubation and mechanical ventilation (MV).^1^ The incidence of VAP varies from 9% to 60% of patients, based on the definition, type of hospital or ICU, study population and levels of antibiotic exposure. VAP is the main cause of nosocomial and acquired infections in ICUs.^2^ Many predisposing factors including age and severity of the underlying diseases are associated with developing VAP. Meanwhile, history of antibiotic exposure and duration of mechanical ventilation are involved.^3^ VAP is also associated with considerable morbidity, including prolonged ICU hospitalization, extended mechanical ventilation and increased costs of hospitalization.^4^ Risk of VAP will be significantly increased up to 1-3% in intubated patients for each day requirement for mechanical ventilation.^5^^,^^6^

The present study aimed to determine ICU related bacteria and their antibiotic sensitivity and risk factors for acquisition of ventilator- associated pneumonia in an intensive care unit (ICU).

## METHODS

***Setting: ***The study was undertaken at the Be'sat teaching hospital in Sanandaj city, Kurdistan province western Iran. This hospital has an ICU with l2 beds.

***Design***: Through a nested case-control study, 149 eligible patients including 48 cases and 101 controls were included. Inclusion criteria were being adult over 15 years, hospitalized and being intubated and mechanically ventilated for more than 48 hours. The study was completed between September 2007 to June 2008.

***Definitions: ***Pneumonia was considered ventilator associated when its onset occurred after 48 hours of mechanical ventilation and it was diagnosed when new, persistent pulmonary infiltrates appeared on chest radiographs along with at least two of following criteria: fever of ≥ 38°C, leukocytosis of 10,000\mm^3^ or more, and purulent respiratory secretions. In cases of clinically suspected pneumonia, endotracheal aspirate (EA) was performed early in the morning; and the diagnosis of VAP was established with a positive quantitative culture (a cut-off point of ≥106 CFU/ ml was considered). To analyze the predisposing factors for development of VAP, the following variables were considered: age, gender, underlying diseases (diabetes mellitus, COPD and infection on admission), diagnosis besides total ICU hospitalization, antibiotic therapy and length of mechanical ventilation.

***Statistical Analysis: ***Univariate analysis was used to compare variables for the outcomes of interest. Continuous data were compared using the Student’s *t *test. Either χ^2^ or Fisher’s exact tests were used to compare categorical variables. A multivariate analysis was also performed using multiple logistic regressions with stepwise approach. All P values lower than or equal to 0.05 were considered statistically significant.

The study was approved by the Kurdistan University of Medical Sciences Ethical Committee. An informed consent was obtained from each patient.

## RESULTS

Of the 149 samples from endotracheal tube of 183 patients who were hospitalized in the adult ICU, 48 VAP cases were diagnosed with an incidence rate of 32.2%. Microorganisms responsible of VAP isolated from endotracheal tube were essentially *Enterobacteriacae* 39 (81.3%) with the head *Klebsiella spp* and isolates of *Acintobacter spp,* *Staphylococcus Epidermidis*, *Pseudomonas spp*. and *Staphylococcus aureus* were 3, 3, 2, and 1 respectively. The maximum and minimum of antibiotic resistance against gram negative bacteria were 93.3% for *Cefalotin* and 50% for *Amikacin*.

Mean duration of hospitalization ± standard deviation was 23.39 ± 10.16 days. The mean interval between intubation, admission to the ICU, hospital admission and VAP identification were 3.7, 4.1, and 6.5 days, respectively. The main risk factors of VAP were mechanical ventilation (Adjusted OR: 1.55, 95% CI: 1.37-1.74), history of antibiotic exposure (AOR: 8.92, CI: 1.16- 66.66) and fever (AOR: 3.11, CI: 1.22- 7.93) as shown in Table-I.

**Table-I:** Risk factors of acquisition of ventilator-associated pneumonia in an adult ICU in Kurdistan-Iran

***VAP, n (%) ***	***No *** *(Controls)*	***Yes *** *(Cases)*	***P value***
*Patient's gender*			
Male	94 (70.7)	39 (29.3)	0.13
Female	41 (82)	9 (18)	
*Antibiotic exposure*			
Yes	116 (71.2)	47 (28.8)	0.03
No	19(95)	1(5)	
*Fever*			
Yes	93 (66.4)	47 (33.6)	<0.000
No	42(97.7)	1 (2.3)	
*Duration of hospitalization*			
Yes	28(100)	0	<0.000
No	107(69)	48(31)	
*Age groups (year)*			
15-20	14(87.5)	2(12.5)	0.42
21-40	64(70.3)	27(29.7)	
41-60	21(67.7)	10(32.3)	
61-80	30(76.9)	9 (23.1)	

**VAP** *: Ventilator-associated Pneumonia*

## DISCUSSION

The present study seems to be representative based on the nested case-control design and considerable sample size. The incidence rate of VAP was estimated to be 26.23 per 100 admitted patients and 32.2 per 100 ventilated patients. A corresponding rate of 7.16 per 100 admitted patients and 50 per 100 ventilated patients have already been reported in Senegal.^7^ An incidence rate of 22.6 per 1000 ventilator days has also been reported in Istanbul, Tureky.^8^ An Italian study has reported an incidence rate of 36.9 per1000 ventilator days.^9^

The mean intervals between intubation, admission to the ICU, hospital admission and the VAP identification in the present study were 3.7, 4.1, and 6.5 days respectively. This is comparable with a recent report of 3.3, 4.5, and 5.4 days in the United State.^10^

In the present study, the infection was polymicrobial. In a recent study conducted by Combes and colleagues, the ICU mortality rate, duration of MV and rate of infection relapse were not significantly affected by monomicrobial and polymicrobial VAP.^11^

The independent significant risk factors for acquisition of VAP in present study were mechanical ventilation, antibiotic exposure, duration of hospitalization and fever. These findings are consistent with a Nicaraguan study in which fever and duration of hospitalization were associated with acquisition of VAP^12^, and with te Baran's study in which nosocomial infection was related to duration of hospitalization.^13^ Earlier similar findings have been reported.^14^^,^^15^ However, unlike the present study, it has shown that the independent risk factors for late versus early VAP acquired in ICU was advanced age.^16^

A recent case-control study showed that inappropriate initial antibiotic treatment was independently associated with relapse of VAP.^6^ Adequacy of antimicrobial therapy, duration of mechanical ventilation and duration of ICU hospitalization have already been reported to be associated with VAP.^17^^-^^21^

In conclusion, VAP is significantly related to ICU hospitalization, mechanical ventilation and history of antibiotics consumption. The maximum and minimum of antibiotic resistance against gram negative bacteria were 93.3% for *Cefalotin* and 50% for *Amikacin.*

## Notes

***Grant Support:*** Deputy of Research Affairs, Kurdistan University of Medical Sciences, Iran.
